# EHDCD: An Edge Enhanced Hierarchical Dual Gated Network for Forest-Cropland Change Detection

**DOI:** 10.3390/s26041175

**Published:** 2026-02-11

**Authors:** Tingting Zhao, Yicong Sun, Xia Yu, Liqian Zhang, Quanping Zhang, Yunli Bai

**Affiliations:** 1 College of Computer and Information Engineering, Inner Mongolia Agricultural University, Hohhot 010018, China; 18734950609@emails.imau.edu.cn (T.Z.); syc20005885@163.com (Y.S.); yuxia@imau.edu.cn (X.Y.); zhanglq@imau.edu.cn (L.Z.); zqp984527172@163.com (Q.Z.); 2 Inner Mongolia Autonomous Region Key Laboratory of Big Data Research and Application of Agriculture and Animal Husbandry, Hohhot 010018, China

**Keywords:** change detection, dual gated feature compensation, edge enhancement, forest-cropland, hierarchical adaptation

## Abstract

Aiming at the differences in spatial spectral attributes between forested land and cultivated land on remote sensing images, and the deficiencies of existing remote sensing change detection methods that are difficult to capture fine edge structures and distinguish pseudo changes, this paper introduces an Edge Enhanced Hierarchical Dual Gated Change Detection (EHDCD) model for forested land and cultivated land, aiming to meet the demand for representing the complex features of these two land types. The model designs an Edge Enhanced Channel Attention Module (EECA) to strengthen the edge recognition ability and suppress the noise interference; proposes a High-Low Level Dynamic Adaptation Strategy (HiLo) to realize the balanced expression of detail information and semantic features; and constructs a Dual Gated Feature Compensation Module (DGFM) to effectively reduce the misdetection rate of change detection. Experiments show that the F1 scores of the model on the self-constructed forest and agricultural dataset FC-CD and public datasets CLCD and SYSU-CD reach 89.06%, 83.37%, and 85.06%, respectively, which can more accurately support the dynamic monitoring applications of forest land and cropland.

## 1. Introduction

Remote sensing change detection constitutes a key technology for monitoring terrestrial dynamics and is critically applied in forest vegetation monitoring [1], land-cover analysis [2,3], ecological conservation [4], and agricultural trend assessment [5] while providing essential data support for regional sustainable-development decision-making [6,7,8]. Given the central roles of forest land and cropland in ecological and food security, precise dynamic monitoring is of strategic importance for resource protection, red-line regulation, and responses to climate change [9,10,11]. With the popularization of high-resolution remote sensing images, change detection technology has gradually developed towards intelligence and refinement [12], and its core challenge lies in how to balance the feature characterization capability and computational efficiency to adapt to the detection needs of complex surface scenes.

In terms of technical evolution, early studies were primarily based on CNNs. For instance, FC-EF, FC-Siam-Diff, and FC-Siam-Conc [13], together with SNUNet [14], employed Siamese fully convolutional architectures to extract local features, yet they were constrained by limited receptive fields that hindered the capture of large-scale change associations; HANet [15] introduced row-column attention to enhance global modeling, yet efficiency remained insufficient when processing large-scale imagery.

To overcome the bottleneck in global-dependency modeling, Transformers were introduced into the domain of change detection [16,17]. BIT [18] modeled spatiotemporal context via semantic tokens, ChangeStar [19] introduced single-temporal supervision, and ChangeFormer [20] and SwinSUNet [21] adopted hierarchical or pure transformer architectures to improve accuracy. However, the self-attention mechanism induces computational complexity that scales quadratically with image size, rendering these methods less suitable for continuous monitoring of large-area forest and cropland.

In recent years, the Mamba architecture, based on state-space models, with linear computational complexity and strong long-sequence modeling capabilities, has provided new solutions for remote sensing change detection [22,23,24]. ChangeMamba [25] captured multi-directional context through a cross-scan mechanism; RS-Mamba [26] designed omnidirectional scanning to accommodate the multi-directionality of remote sensing imagery. AtrousMamba [27] and CDMamba [28] optimized detail extraction via dilated (atrous) scanning and local–global fusion, respectively.

However, these methods are mostly designed for rigid targets such as buildings and roads, and do not fully consider the unique attributes of forested and cropland: (1) fragmented edge structures, wherein irregular boundaries such as forest edges and field ridges are easily smoothed or lost in feature extraction; and (2) pseudo-change interference, in which spectral fluctuations and shadowing are easy to be misdiagnosed or difficult to be peeled off, leading to pseudo-change labeling. Existing methods lack a targeted mechanism for the above features, resulting in frequent problems such as edge blurring and pseudo-change misdetection, which restricts detection accuracy and application reliability.

To this end, an Edge Enhanced Hierarchical Dual Gated Change Detection (EHDCD) network tailored to forest–cropland scenarios is proposed; Our main contributions are as follows:

(1) Design the Edge Enhanced Channel Attention Module (EECA), which dynamically correlates the edge features with the importance of the channel through adaptive edge detection and channel weight calibration, and suppresses the semantic noise while enhancing the fine edge capture;
(2) The High–Low Level Dynamic Adaptation Strategy (HiLo), working in concert with EECA, adaptively adjusts the compression ratio and edge thresholds according to the difference between the shallow and deep features of the encoder, and realizes the balance between detail retention and semantic focus;
(3) Construct a Dual Gated Feature Compensation Module (DGFM), which works in tandem with the dual-path gating mechanism to screen redundant features introduced by spectral fluctuations and shadow interference, and to strengthen the response signals in the real change region to reduce pseudo-change false detections.

The thesis is structured as follows: Section 2 introduces the related work; Section 3 details the model design; Section 4 presents the experimental results with an analytical discussion of the model advantages and performance efficiencies; and Section 5 summarizes the core contributions and values and looks forward to future research directions.

## 2. Related Work

### 2.1. SSM

The state space model (SSM) [22,29,30] integrates the classical state-space framework from control theory with modern deep learning. Input sequences are mapped to hidden states, from which outputs are generated, thereby capturing long-range dependencies while maintaining computational efficiency.

The mathematics of SSM is the state and observation equations. Given an input sequence x(t), the continuous-time formulation is expressed in (1):

(1) $$\left\{\begin{array}{c} h^{\prime }(t)=Ah(t)+Bx(t)\\ y(t)=Ch(t)+Dx(t) \end{array}\right.$$

where  h(t) is the hidden state, A, B, C, and D are the state transfer, input, output, and direct connection matrices, respectively. Model parameters are optimized via gradient descent to learn the sequence dynamics.

In order to adapt discrete sequence data, SSM needs to be discretized by methods such as Zero Order Holding (ZOH), which transforms the continuous state equations, and is formulated as (2):

(2) $$\left\{\begin{array}{c} h_{t}=\bar{A}h_{t-1}+\bar{B}x_{t}\\ y_{t}=Ch_{t}+Dx_{t} \end{array}\right.$$

where $\bar{A}$ and $\bar{B}$ are the discretized parameters to ensure that the model can directly process sampled data and maintain numerical stability.

The core advantage of SSM is the linear computational complexity O(N), which avoids explicit storage of historical information through recursive state updating, which is significantly better than the O(N^2^) of Transformers, while retaining the parallelism of CNN and the temporal modeling capabilities of RNNs [31,32]. However, the traditional SSM parameters are not flexible enough to capture complex nonlinear patterns and rapidly changing sequence information, and the ability to express long sequences of complex tasks is limited.

To overcome these limitations, researchers proposed S4 [22], which achieves linear complexity and expression capability enhancement through matrix diagonalization, HiPPO initialization, and other constraints, and becomes an important foundation for long sequence modeling. Subsequent variants such as S5 [33] further expand its application scenarios and lay the technical foundation for the development of models such as Mamba.

### 2.2. Mamba

Mamba [34] is a selective state space model based on the SSM framework; the core breakthrough is the combination of input-dependent dynamic parameters and hardware-aware parallel computing. It preserves the linear complexity advantage of SSM while addressing the limited expressiveness of conventional SSM through an “on-demand compute allocation” mechanism that balances efficiency and accuracy, and makes up for the bottleneck of the complexity of the transformer and the shortcomings of the flexibility of traditional SSM [31].

The core innovation of Mamba is selective SSM, where model parameters change dynamically with inputs to focus on key information. The dynamic time step $\Delta_{t}$ is central to the selectivity mechanism and is defined in (3):

(3) $$\Delta_{t}=\sigma (W_{\Delta }x_{t}+b_{\Delta })$$

where $W_{\Delta }$ and $b_{\Delta }$ are learnable parameters; σ is the sigmoid activation; and $\Delta_{t}\in (0,1)$; larger values indicate tokens of higher importance that require finer-grained state updates. Meanwhile, the state transfer matrix ${\bar{A}}_{t}$ and input matrix ${\bar{B}}_{t}$ are dynamically generated from the input, preserving the recursive SSM formulation while enabling adaptive parameter adjustment. The output mapping is given in (4):

(4) $$y_{t}=C_{t}h_{t}+Dx_{t}$$

where $C_{t}$ is dynamically generated from the input, and D is used to supplement the local input information to improve the short-range dependency modeling capability.

Mamba offers three principal performance advantages: linear complexity adapted to ultra-long sequence tasks, and efficiency and memory friendliness of long sequence scenes are better than those of the transformer [34]; it is extended to the field of computer vision by selective scanning in 2D (SS2D) [35], to break the limitation of the traditional SSM temporal modeling, and it can be equated to the generalized linear attention, which has the same underlying structure as the transformer. There exists an underlying structural commonality that facilitates cross-architecture transfer learning [36,37].

## 3. Methodology

### 3.1. Overall Architecture

**Figure 1:**
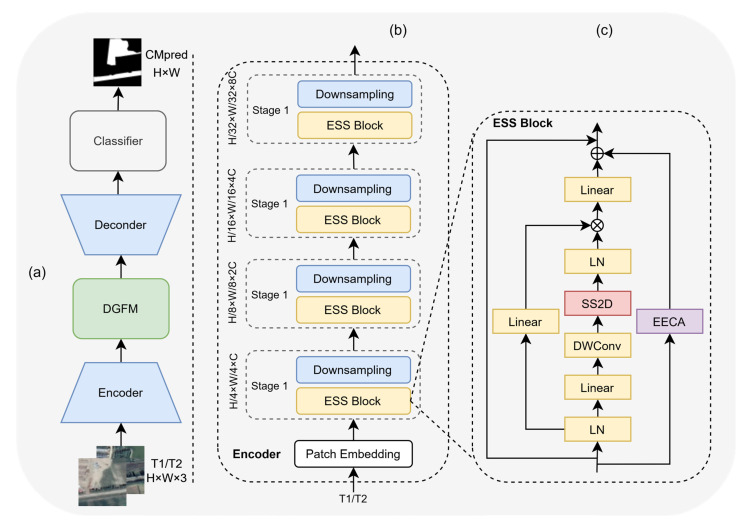
Schematic overview of the proposed method: (**a**) architecture of EHDCD, T1 and T2 denote bi-temporal images, and CMpred denotes the predicted change map; (**b**) encoder composed of ESS blocks; (**c**) architecture of an ESS block incorporating EECA and other components.

As shown in Figure 1, the EHDCD architecture comprises an encoder, an EECA module, a DGFM module, and a decoder.

A pair of bi-temporal remote sensing images—T1 (pre-change) and T2 (post-change), each of size H×W×3. The inputs are processed by an SSM-based encoder, in which a patch embedding layer partitions raw pixel-level imagery into fixed-size patches and performs feature embedding, converting dispersed pixel information into compact embedded representations, and thereby establishing a foundation for subsequent modeling of spatiotemporal correlations. Embedded features are subsequently propagated through four sequential encoder stages (Stages 1–4). Each stage consists of multiple ESS blocks containing SSM branches, captures the temporal and spatial correlation of the features, completes the feature downsampling and dimensionality enhancement at the same time, and finally, the encoder outputs four sets of bi-chronological features with different levels of hierarchical levels: Stage 1 outputs features with channel dimension H/4×W/4×C; Stage 2 outputs H/8×W/8×2C; Stage 3 outputs H/16×W/16×4C; and Stage 4 outputs H/32×W/32×8C. Shallow features retain high-resolution details, whereas deep features emphasize high-level semantics.

Within each ESS block, an EECA module is integrated with a HiLo strategy. Shallow stages employ a low compression ratio and low edge thresholds to enhance detail capture, whereas deep stages use a high compression ratio and high edge thresholds to emphasize semantic associations. This process yields enhanced bi-temporal features whose dimensions match their corresponding stage outputs. The enhanced features are subsequently processed by the DGFM module (detailed in Section 3.4) to generate compensated features. These compensated features directly replace the original encoder features and are fed into the decoder. The decoder restores spatial resolution through bilinear interpolation and processes the four sets of compensated features using a bottom-up, layer-by-layer fusion strategy. This fusion process sequentially performs feature upsampling, pixel-wise addition, and ResBlock smoothing, ultimately generating a unified high-resolution semantic change feature map. Finally, the classifier performs a binary mapping (“change” vs. “no change”) on the feature maps to generate initial predictions. These predictions are obtained by binarizing the two-class probability map (0–1) from the Softmax activation using a fixed threshold of 0.5 (≥0.5 indicates change, <0.5 indicates no change). For large-scale remote sensing images, block-level inference is performed using a 256 × 256 sliding window (stride 128, 50% overlap). Overlapping regions are averaged to fuse predictions and mitigate boundary artifacts. Initial predictions are then upsampled to the original image size via bilinear interpolation, yielding the binary change mask CMpred.

### 3.2. Edge-Enhanced Channel Attention (EECA)

**Figure 2:**
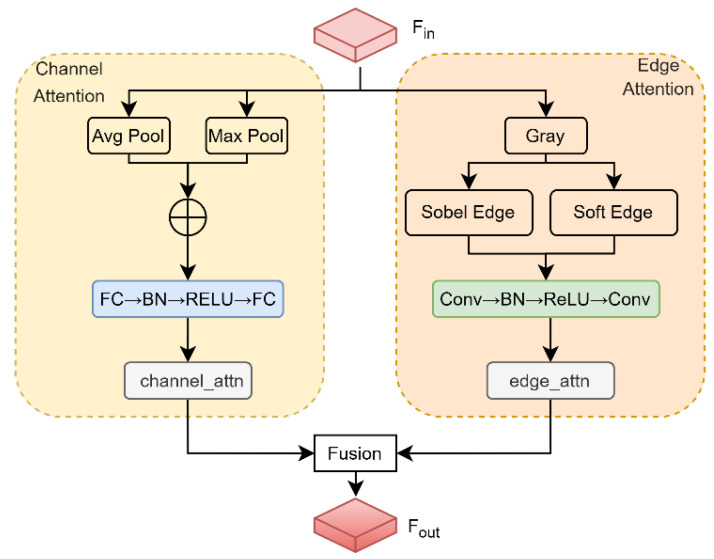
Schematic of the Edge-Enhanced Channel Attention (EECA).

To address the difficulty of simultaneously preserving fine edges and semantic features in forest-cropland change detection, an edge-enhanced channel attention (EECA) module is proposed. As shown in Figure 2, EECA integrates an adaptive edge-detection operator with channel weight calibration to dynamically couple forest-cropland edge features with channel importance. In this way, the capture of fine edges (e.g., field ridges and forest margins) in shallow features is strengthened, while semantic noise in deep features is suppressed, thereby mitigating the imbalance inherent to single-branch attention between detail and semantics. EECA contains a channel attention branch, an edge detection branch, and an adaptive fusion layer.

The channel attention branch focuses on modeling the global importance of each channel of the input feature map. The input feature map $F_{in}$ is passed through global average pooling (Avg Pool) and global max pooling (Max Pool), which capture complementary channel-level statistics: mean and peak responses. The two pooled tensors are concatenated and sequentially processed by a fully connected (FC) layer, batch normalization (BN), a ReLU activation, and a second FC layer; a sigmoid function then produces the channel-attention weights (channel_attn), as given in (5):

(5) channel_attn=σFFC-BN-ReLU-FC(AvgPool(Fin)⊕MaxPool(Fin))

In (5), $F_{\mathrm{FC}-\mathrm{BN}-\mathrm{ReLU}-\mathrm{FC}}$ denotes the FC-BN-ReLU-FC sequence. BN and ReLU together improve numerical stability, and the Sigmoid function constrains the output range to ([0, 1]), helping the model concentrate on semantically important channels.

The edge detection branch extracts edge information from $F_{in}$ and generates associated attention weights to emphasize high-frequency details such as field ridges and forest margins. First, $F_{in}$ is converted to a grayscale map $G=Gray(F_{in})$; a Sobel operator then produces an edge-strength map $E_{sobel}=SobelEdge(G)$; soft thresholding yields $E_{soft}=SoftEdge(G)$. These three maps are concatenated as in (6):

(6) $$F_{concat}=Concat(E_{sobel},E_{soft},G)$$

The concatenated representation is processed by a convolutional subnetwork to generate edge attention weights edge_attn, as defined in (7):

(7) $$edge\_attn=F_{Conv-BN-RELU-Conv}(F_{concat})$$

In (7), $F_{Conv-BN-RELU-Conv}$ denotes the Conv-BN-ReLU-Conv sequence and concat denotes feature concatenation. Range limiting and adaptive thresholding of edge strength improve numerical stability and enable precise edge capture.

An adaptive fusion layer uses a learnable weight α∈(0,1) to blend channel_attn and edge_attn, balancing their contributions and producing composite attention weights, as expressed in (8):

(8) attn=α⋅channel_attn+(1−α)⋅edge_attn

This fusion mechanism adaptively adjusts the allocation between detail and semantic focus according to input content, markedly improving generalization.

The final composite attention (attn) is applied element-wise to Fin and combined with a residual connection to yield $F_{out}$, as shown in (9):

(9) $$F_{out}=attn⊙F_{in}+F_{in}$$

In (9), ⊙ denotes element-wise multiplication. This design amplifies salient features while preserving original information, and the residual path helps prevent gradient explosion.

Training stability is supported in EECA through BN, output range constraints, and residual connections. Its adaptive fusion mechanism and hierarchical parameter configuration enhance feature representation for forest-cropland change detection, enabling simultaneous attention to semantic information and edge detail for more accurate discrimination and extraction.

### 3.3. High-Low Level Dynamic Adaptation Strategy (HiLo)

**Figure 3:**
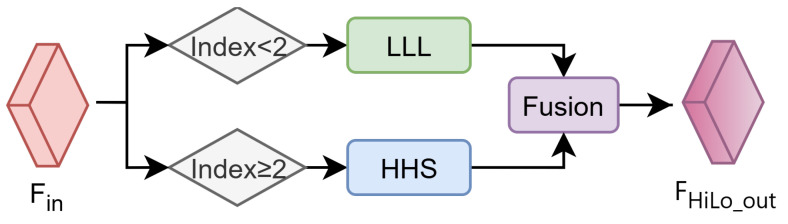
Schematic of the High-Low Level Dynamic Adaptation Strategy (HiLo). LLL denotes the configuration with Low Compression Ratio, Low Edge Threshold, and Large Initial Weight, and HHS denotes the configuration with High Compression Ratio, High Edge Threshold, and Small Initial Weight.

Aiming at the inherent differences in resolution and semantics of features at different levels in the encoder: shallow features are rich in details, but weak in semantics, and deep features are strong in semantics but lose in details, the traditional method uses fixed parameter configurations, which makes it difficult to achieve global optimization. To this end, the High-Low Level Dynamic Adaptation Strategy (HiLo) is proposed. As shown in Figure 3, HiLo takes the current stage features $F_{in}$ as input and applies layered parameter configuration and learnable weight control to jointly optimize shallow-level detail and deep-level semantics. HiLo sets an initial compression ratio of 16 and an edge threshold of 0.15, employing a hierarchical linear scaling strategy: shallow levels (index < 2) use a threshold of 0.075 (0.5 times the initial value) while maintaining a 16x compression rate; Deep levels (index ≥ 2) use a threshold of 0.225 (1.5 times the initial value) and an 8x compression rate (half the initial value). The core idea is to dynamically adjust key EECA parameters and attention weights for $F_{in}$ according to the encoder stage index, and then fuse them to produce stage-adapted outputs $F_{HiLo\_out}$.

When the module is in the shallow stage (Index < 2), a combination of low compression ratio (16) and low edge threshold (0.075) configuration is used to address the dominance of detail edges in high-resolution features. The low compression ratio preserves subtle inter-channel differences and increases the sensitivity of channel attention to detail-related channels. The low edge threshold activates a larger set of weak yet critical edge responses, directing EECA toward more effective extraction of fine edge structures. In addition, the initial fusion weight is set to a relatively large value to amplify the contribution of edge detail during attention fusion, ensuring that shallow stages prioritize discriminative structures and preventing detail loss caused by excessive compression or overly strict thresholding.

When the module is in the deep stage (Index ≥ 2), the configuration scheme of High Compression Ratio (8) and High Edge Threshold (0.225) is adopted for the dominance of semantic information in the low-resolution features. The high compression ratio reduces redundant channels, concentrates representation capacity on those most contributive to semantic classification, and suppresses noise in semantically ambiguous low-resolution regions. The high edge threshold filters pseudo-edges introduced by downsampling blur, retaining only salient semantics-associated edges. In addition, the initial fusion weight is set to a relatively small value to limit interference from edge detail with semantically dominant features, ensuring that deep stages preferentially enhance semantic consistency.

HiLo achieves dynamic tuning through learnable attention fusion weights. This weight is co-optimized with the model parameters during the training process and can automatically adjust the balance ratio between details and semantics according to the specific content of the input image. The synergistic mechanism between layered parameter configuration and dynamic weight regulation enables the EECA module to adapt to stage-dependent feature characteristics: strengthening fine edge detection in shallow high-resolution stages and focusing on global semantic expression in deep low-resolution stages. HiLo is deeply integrated with the edge-channel joint modeling mechanism of EECA, forming the core feature-enhancement component of EHDCD. By differentially adapting to features at distinct encoder levels, the contribution of detail and semantic information is effectively balanced, providing a feature foundation for subsequent temporal difference modeling and pseudo-change suppression, thereby improving change-detection performance in complex surface scenarios.

### 3.4. Dual-Gated Compensation Module (DGFM)

**Figure 4:**
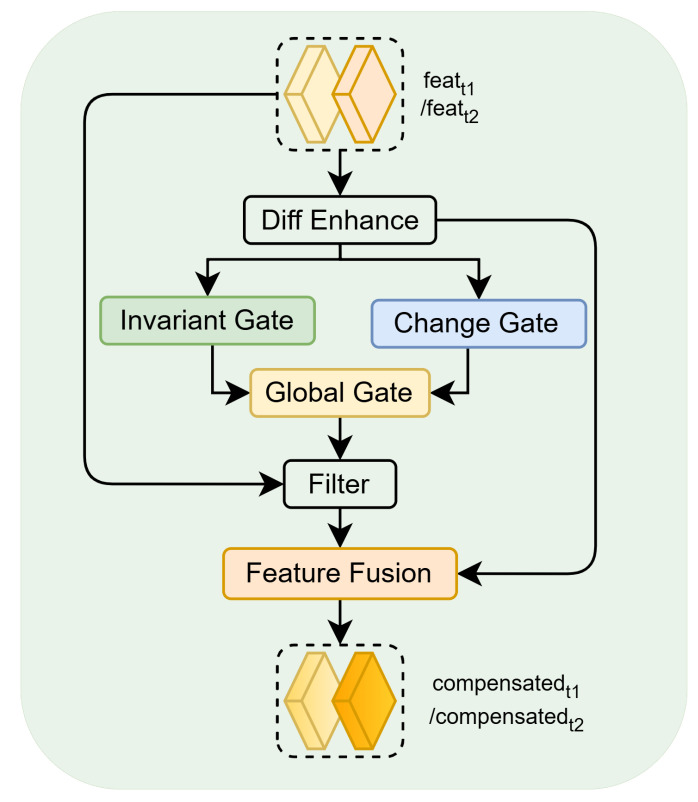
Schematic of the Dual-Gated Compensation Module (DGFM).

In change detection for forests and croplands, precise localization of true change regions in bi-temporal imagery and suppression of pseudo-changes induced by non-change factors (e.g., shadows and occlusions) are required. Traditional approaches rely on simple difference operations or fixed-weight feature fusion, making it difficult to dynamically balance the enhancement of change signals with the suppression of redundant information. To this end, a Dual-Gated Compensation Module (DGFM) is proposed, as illustrated in Figure 4. The module employs a dual-gating mechanism to dynamically select key features, a difference-enhancing convolution to strengthen change signals, and learnable weights to optimize fusion ratios, thereby effectively filtering bi-temporal redundancy and precisely enhancing change signals.

The DGFM module takes bi-temporal feature maps $feat_{t1}$ and $feat_{t2}$ as input, and outputs the compensated bi-temporal features $compensated_{t1}$ and $compensated_{t2}$. The core design principle is to balance the contributions of change and no-change features via dynamic gating weights, highlight key change signals using enhanced difference features, and preserve fine details within change regions through a feature compensation mechanism.

The module first computes the absolute difference map of the bi-temporal features, which directly reflects per-pixel spectral change intensity, as shown in Equation (10):

(10) $$D=\left|feat_{t1}-feat_{t2}\right|$$

To enhance the salience of key change signals in the difference map, a difference-enhancing convolution is introduced. This convolution extracts local context through a combination of depthwise and pointwise convolutions and introduces nonlinearity via batch normalization and ReLU, as shown in Equation (11):

(11) $$Diff\;Enhance=BN\left(ReLU\left(PointConv_{1\times 1}\left(DepthConv_{3\times 3}\left(D\right)\right)\right)\right)$$

Here, $DepthConv_{3\times 3}$ denotes a depthwise convolution with a 3×3 kernel, and $PointConv_{1\times 1}$ denotes a pointwise 1×1 convolution. To mitigate overfitting, dropout with a probability of 0.1 is applied to the enhanced difference features $D_{\mathrm{enh}}$. Its range is maintained in [0,+∞), and the magnitude directly corresponds to the confidence of the bi-temporal difference.

Based on the enhanced difference features, two parallel branches—change gating and no-change gating—are designed to focus on true change regions and stable no-change regions, thereby dynamically generating feature-selection weights. The change-gating branch takes Diff Enhance as input and produces the change-region weight $G_{change}$ via two convolutional layers, while the no-change-gating branch takes $1-D_{\mathrm{enh}}$ as input and adopts the same architecture to produce the no-change-region weight $G_{\mathrm{invariant}}$, as shown in Equation (12):

(12) $$\left\{\begin{array}{c} G_{change}=Conv_{2}(ReLU(Conv_{1}(Diff\;Enhance)))\\ G_{invariant}=Conv_{2}(ReLU(Conv_{1}(1-Diff\;Enhance))) \end{array}\right.$$

To avoid over- or under-suppression caused by fixed weights, learnable gating weights α and β are introduced; after sigmoid normalization, they dynamically balance the contributions of the two branches to yield the global gating weight $G_{\mathrm{gate}}$, as shown in Equation (13):

(13) $$G_{gate}=\sigma (\alpha \cdot G_{change}+\beta \cdot G_{in\mathrm{var}iant}+b_{g})\;$$

Here, $b_{g}$ is a gating bias that prevents the gating weight from approaching zero and thus fully suppressing features. $G_{\mathrm{gate}}$ fuses local difference responses with global learned preferences, enabling adaptive balancing of contributions from change and no-change features.

Based on the dynamic gating weight $G_{\mathrm{gate}}$, redundancy in the original bi-temporal features is filtered: channel-wise multiplication suppresses redundant information in low-weight regions and preserves key features in high-weight regions, thereby effectively reducing pseudo-change interference. To supplement detail in true change regions, the filtered features are summed with the enhanced difference signal to obtain the outputs $compensated_{t1}$ and $compensated_{t2}$, as shown in Equation (14):

(14) $$\left\{\begin{array}{c} compensated_{t1}=(feat_{t1}⊙G_{gate})+Diff\;Enhance\\ compensated_{t2}=(feat_{t2}⊙G_{gate})+Diff\;Enhance \end{array}\right.$$

Finally, batch normalization is applied to the compensated features to further stabilize their value distribution, thereby providing a consistent input to subsequent network layers.

Pseudo-change interference is effectively suppressed by the DGFM module through the synergistic action of dual gating, difference enhancement, and feature compensation. Its core contribution lies in markedly improving the discrimination of true change signals and the robustness of feature representation, thereby providing essential support for high-precision temporal change modeling in forests and croplands.

### 3.5. Loss Function

To effectively train the change detection model and address class imbalance between foreground and background, a hybrid loss termed Hybrid Loss is adopted, combining cross-entropy loss and Lovász-Softmax loss [38]. Integrating cross-entropy loss with Lovász-Softmax loss helps mitigate the severe class imbalance between changed and unchanged pixels in forest and cropland change detection tasks, overcoming the limitations of single loss functions.

The cross-entropy loss $L_{CE}$ is computed as in Equation (15):

(15) $$L_{CE}=-\frac{1}{N}\sum_{i=1}^{N}\sum_{C=i}^{C}y_{i,C}\log(p_{i,C})$$

Here,  N denotes the total number of pixels in a batch, C denotes the number of classes (with C=2 in this study), $y_{i,C}$ is the ground-truth label of the pixel i for class C, and $p_{i,C}$ is the predicted probability that pixel i belongs to the class C. This loss provides per-pixel classification gradients to the model.

However, using only cross-entropy loss biases the model toward the majority no-change regions, thereby degrading detection performance on change regions. To mitigate this issue, the Lovász-Softmax loss is introduced. This loss is a convex surrogate of the IoU metric and directly optimizes the intersection-over-union in segmentation; its reduced sensitivity to class imbalance allows a stronger focus on improving accuracy in change regions.

The final loss (L) is the weighted sum of the two components, as shown in Equation (16):

(16) $$L=L_{CE}+\alpha \cdot L_{Lov}$$

## 4. Experiments

### 4.1. Datasets

#### 4.1.1. FC-CD

**Figure 5:**
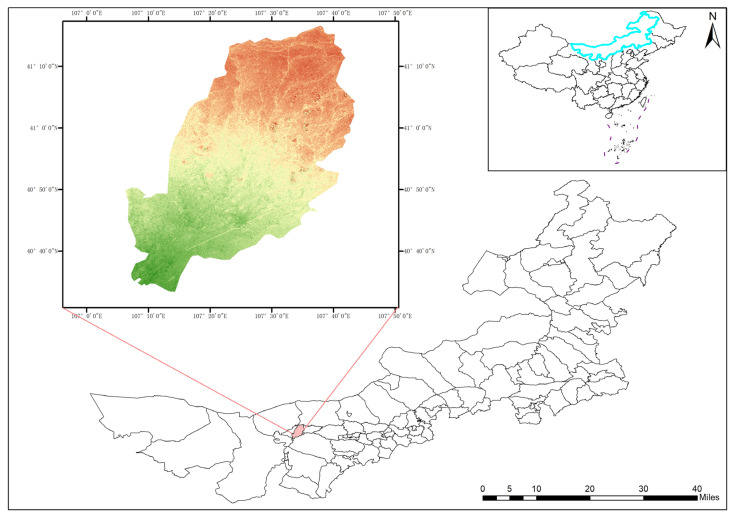
Overview of the Xinhua Forest Farm as the study area. The color gradient from green to red represents elevation from low to high.

To enhance the specificity of the study, a binary remote sensing change-detection dataset for forests and croplands, termed the Forest-Cropland Change Detection Dataset (FC-CD), is constructed, focusing on changes in forests and croplands. As shown in Figure 5, FC-CD is centered on the Xinhua Forest Farm in Linhe District, Bayannur City, Inner Mongolia, China. The forest farm is located in the core area of the Hetao Plain, above the bent section of the Yellow River; it faces the Ordos Plateau across the river to the south, borders the Yin Mountains to the north, and adjoins the Urad Grassland to the east. Its geographic coordinates are 107°06′–107°44′ E and 40°34′–41°17′ N.

The forest farm covers an area of approximately 34.7 km^2^ and comprises four sub-farms: Machangdi, Banchen, Xinsheng, and Hada. It is an important area of the “Three-North” Shelterbelt Project and possesses substantial ecological value. The study area consists of forest land, cultivated land, buildings, waters, and other types. Forests and croplands are selected as the target classes for change detection. The area exhibits typical characteristics of sandy-land management and ecological restoration, making it a suitable site for studying changes in forests and croplands.

The FC-CD dataset is sourced from Google Earth remote sensing imagery. Two images acquired in August 2017 and August 2024 are selected, with a spatial resolution of approximately 1.6 m and three visible bands: red (620–670 nm), green (520–570 nm), and blue (450–500 nm). All labels are manually annotated through visual interpretation. The images are cropped into 256 × 256 pixel patches, yielding 945 pairs of initial samples. For data augmentation, image rotation, translation, brightness adjustment, and noise injection are applied, resulting in 1616 image pairs. For the labeled image pairs, this paper adopts a stratified random sampling method and divides the data set into a training set, testing set, and validation set in the ratio of 7:2:1, which are used for model training, performance evaluation, and validation, respectively.

#### 4.1.2. CLCD

CLCD [39] is a remote sensing dataset focused on cropland change detection, comprising 600 pairs of bi-temporal agricultural images. The images were acquired by the Gaofen-2 satellite in Guangdong Province, China, in 2017 and 2019. Each sample consists of two 512×512 images, with a spatial resolution of 0.5–2.0 m. The annotated primary change types include buildings, roads, lakes, and bare soil. The dataset is split into training (360 pairs), validation (120 pairs), and test (120 pairs) sets.

#### 4.1.3. SYSU-CD

SYSU-CD [40] is a high-resolution, bi-temporal change-detection dataset comprising 20,000 pairs of ortho-rectified aerial images with a spatial resolution of 0.5 m and a size of 256×256. The imagery was primarily acquired in the Hong Kong region of China, spanning 2007 to 2014. The dataset covers diverse land-cover objects and change scenarios, including urban construction, suburban expansion, foundation works, vegetation change, road expansion, and marine engineering. Following a 6:2:2 split, the 20,000 pairs are divided into 12,000 training pairs, 4000 validation pairs, and 4000 test pairs.

### 4.2. Experimental Setup

AdamW [41] is employed during training, with an initial learning rate of $1\times 10^{-4}$, a batch size of 8, and a weight-decay coefficient of $5\times 10^{-4}$ to mitigate overfitting and enhance generalization. To further prevent late-stage overfitting and reduce ineffective iterations, an early-stopping mechanism is introduced to dynamically terminate training. The model is implemented in the PyTorch (v2.1) deep learning framework and trained and evaluated on an NVIDIA Tesla V100 GPU.

### 4.3. Evaluation Metrics

In order to comprehensively evaluate the performance of the model in the change detection task, this paper selects five core evaluation metrics from different dimensions, including Recall (Rec), Precision (Pre), Overall Accuracy (OA), F1-Score (F1), and Intersection over Union (IoU). Specifically, recall measures the class-wise completeness of foreground detection; precision quantifies the accuracy of predictions; overall accuracy reflects the global correctness of classification over all pixels (foreground and background). The F1-score, as the harmonic mean of recall and precision, balances the trade-off between missed detections and false alarms, providing a balanced assessment of performance. IoU, as a core metric in segmentation, quantifies the regional overlap between the predicted and ground-truth foreground regions. The formulas for each index are as follows (17)–(21):

(17) Rec=TPTP+FN

(18) $$\mathrm{Pr}e=\frac{TP}{TP+FP}$$

(19) $$OA=\frac{TP+TN}{TP+TN+FP+FN}$$

(20) F1=2×Pre×RecPre+Rec

(21) $$IoU=\frac{TP}{TP+FP+FN}$$

### 4.4. Comparison with Different Models

To evaluate the effectiveness and superiority of the proposed EHDCD method, ten classical and contemporary change detection networks are employed as baselines, covering three representative technical paradigms in remote-sensing change detection: CNN-based, transformer-based, and Mamba-based methods.

Specifically, FC-EF, FC-Siam-Diff, and FC-Siam-Conc [13] are representative fully convolutional network—based methods, and their core design of cross-temporal phase feature fusion, difference operation, and other core designs lay an important foundation for subsequent research; SNUNet [14] introduces multi-scale feature aggregation and attention mechanisms, thereby enhancing feature extraction in CNN architectures for complex land-cover change detection. Among transformer-based approaches, BIT [18] and Changer [42] refine the use of self-attention for remote-sensing change detection, while ChangerFormer [20] integrates the strengths of transformers and CNNs to improve the joint modeling of global context and local features. ChangeMamba [25] and RS-Mamba [26] represent early explorations of the Mamba architecture in this field, establishing efficient cross-temporal change association modeling. EHDCD is the method proposed in this study.

Experiments are conducted on the public datasets CLCD and SYSU-CD, as well as the self-built dataset FC-CD. Using the core metrics, Rec, Pre, OA, F1, and IoU, performance differences among models are quantified and illustrated across scenarios such as cropland and forestland. The test results are reported in Table 1, Table 2 and Table 3.

On the CLCD dataset, Mamba-based models perform markedly better than traditional CNN-based and transformer-based models. Although the IoU of EHDCD is slightly lower than that of RS-Mamba, the other core metrics are superior overall; the F1-score improves by 10.87% over RS-Mamba and by 1.80% over ChangeMamba, demonstrating a clear advantage in change detection for general remote-sensing scenarios.

The complex scene of the SYSU-CD dataset puts higher requirements on the model’s ability to capture changes. EHDCD achieves the highest Rec, OA, and F1, and the second-highest Pre and IoU, with the F1-score 2.23% higher than ChangeMamba, demonstrating the stable detection ability in complex scenes.

**Figure 6:**
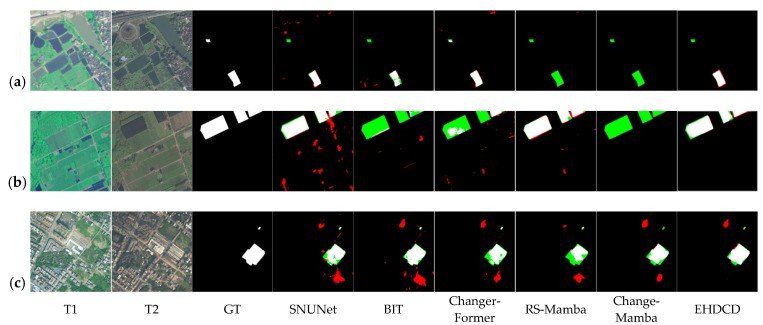
Visualization results of different methods on the CLCD test set. White: TP, black: TN, red: FP, green: FN. (**a**) Cropland change; (**b**) Cropland change; (**c**) Building change.

**Figure 7:**
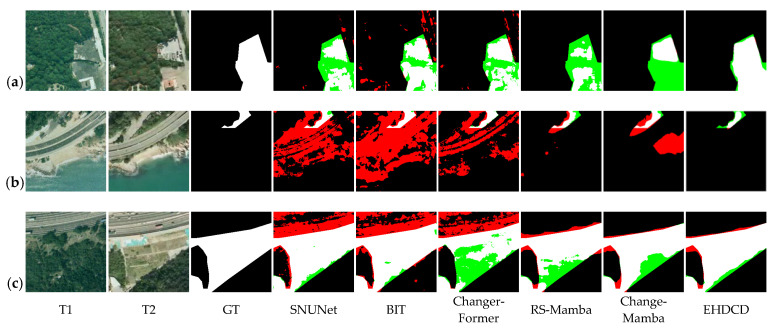
Visualization results of different methods on the SYSU-CD test set. White: TP, black: TN, red: FP, green: FN. (**a**) Green space change; (**b**) Forestland change; (**c**) Forestland change.

**Figure 8:**
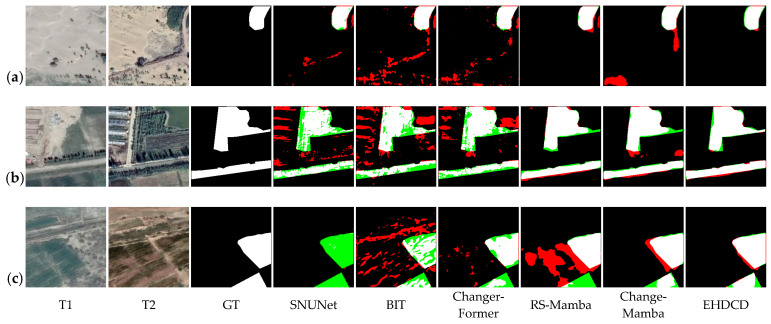
Visualization results of different methods on the FC-CD test set. White: TP, black: TN, red: FP, green: FN. (**a**) Forestland change; (**b**) Forestland change; (**c**) Cropland change.

To further validate the actual detection efficacy of EHDCD, visual analyses of the predicted results were conducted on the CLCD, SYSU-CD, and FC-CD test sets, as shown in Figure 6, Figure 7 and Figure 8. The analysis shows that EHDCD achieves the highest agreement between the predicted results and the ground-truth labels. Specifically, the model provides a more complete reconstruction of fine and fragmented edges while maintaining more effective suppression of pseudo-change interference, resulting in more balanced overall performance.

In terms of edge-detail capture, EHDCD demonstrates strong adaptability to the fine, fragmented linear boundaries characteristic of forestland and cropland. Taking the irregular boundary of the cultivated field ridge in Figure 6a as an example, the true-positive region in the EHDCD prediction results matches better with the real changing contour of the ridge, and the edge lines remain coherent; in the forest edge scene in Figure 8a, its prediction results also cover the natural transition boundary of the forest edge better, and the overlap between the edge pixels and the real labels is better than that of the comparative model; and in the woodland reduction scene in Figure 7c, its prediction results are also more accurate in portraying the edges of the woodland reduction area. This improvement is primarily attributed to EECA, which reduces edge over-diffusion observed in SNUNet and BIT, and mitigates edge miss-detections in ChangerFormer.

In summary, the visualization results and quantitative metrics provide complementary validation: EHDCD outperforms the baseline models in fine-edge depiction and pseudo-change suppression. In the FC-CD dataset specialized for forestland and cropland, its adaptability to complex object boundaries and small-scale changes is particularly pronounced.

### 4.5. Ablation Study

A series of ablation experiments were designed to validate the effectiveness and synergy of EECA, HiLo, and DGFM proposed in this study. To ensure the fairness of experimental comparisons and the validity of results, all comparison models in the ablation experiments employ fully consistent hyperparameter settings. As summarized in Table 4, five model variants were built on the MambaCD-Small baseline: Base, Base + EECA, Base + EECA + HiLo, Base + DGFM, and EHDCD.

Ablation experiments were conducted on the CLCD and FC-CD datasets, using Rec, OA, F1, and IoU as evaluation metrics. The results indicate a stepwise improvement across model variants. On CLCD, the base model achieved an F1 of 81.57% and IoU of 68.88%, while EHDCD improved these to an F1 of 83.50% and IoU of 71.68%. On FC-CD, the Base model achieved F1 of 86.01% and IoU of 75.52%, whereas EHDCD increased them to F1 of 89.26% and IoU of 80.61%.

Each module demonstrates independent effectiveness on the basis of MambaCD small: EECA strengthened edge-channel feature associations, leading to an F1 improvement of 1.68% on FC-CD and enhancing the capture of fine structures such as field ridges and forest edges. The HiLo strategy dynamically adjusted optimization parameters across encoder stages, yielding an F1 improvement of 0.45% on FC-CD and improving the joint representation of shallow detail features and deep semantic features. The DGFM module, introduced without altering the base architecture, increased F1 by 2.07% on FC-CD, improved the localization accuracy of change regions, and filtered pseudo changes caused by spectral fluctuations and tree-shade occlusions. Synergistic effects are more pronounced in EHDCD; relative to single-module variants, the integrated model achieved an F1 improvement of 3.25% on FC-CD, reflecting complementary advantages among components while balancing architectural efficiency and detection accuracy. It is worth noting that the performance gains of each module on FC-CD exceed those on CLCD, indicating that the extended module design is better suited to forest–cropland scenarios characterized by complex edge structures and frequent pseudo changes.

In summary, the EECA, HiLo, and DGFM modules each independently improve performance metrics, with DGFM contributing most to IoU and EECA yielding clear gains in F1. The three modules synergize to make the model performance optimal, and the module design has a stronger adaptability to the forested and arable land scenarios, which effectively solves the core detection problem of the base model in this scenario.

### 4.6. Efficiency Analysis

To evaluate the computational efficiency and practical value of the EHDCD model, a comparative analysis was conducted with mainstream change detection models using Params and FLOPs as the primary metrics. This assessment aims to determine whether the model maintains reasonable computational overhead while achieving greater accuracy. The comparison results of model efficiency metrics are presented in Table 5.

In terms of parameter sizes, traditional CNN-based models and transformer-based models exhibit smaller parameter counts, reflecting their lightweight characteristics. In contrast, Mamba-based models generally possess larger parameter sizes; for example, RS-Mamba and ChangeMamba contain 51.95 M and 54.00 M parameters, respectively, while the proposed EHDCD model comprises 57.81 M parameters. This slight increase is primarily attributed to learnable parameters introduced by the newly added EACA and DGFM modules. Nevertheless, the growth is limited to within 7%, thereby maintaining the parameter efficiency characteristic of the Mamba architecture.

With respect to computational complexity, certain transformer models and FC-series models remain at relatively low levels. Among Mamba-based models, RS-Mamba and ChangeMamba entail 22.82 G and 30.92 G FLOPs, respectively, whereas the EHDCD model demonstrates 31.89 G FLOPs—representing only a 3.1% increase relative to ChangeMamba. This limited increase is attributed to the inherent linear complexity advantage of the Mamba architecture, which effectively controls the computational cost introduced by the new modules.

In summary, the EHDCD model achieves a balance between accuracy and efficiency while retaining the efficient computational characteristics of Mamba-based models. Although the model’s parameter size and computational complexity are higher than those of traditional CNN and certain transformer models, this increased overhead results in a substantial improvement in woodland-to-cropland change detection accuracy. Compared to other Mamba-based models, the increases in efficiency metrics are justified by greater accuracy gains, demonstrating a favorable trade-off. Therefore, the EHDCD model is well-suited to meet the computational efficiency requirements of large-scale woodland-to-cropland monitoring in practical scenarios.

## 5. Conclusions

In this study, the EHDCD model is proposed to address the core challenges of fine-grained edge detection and susceptibility to pseudo-change interference in woodland-to-cropland change detection, based on the Mamba architecture with linear computational complexity. Targeted optimization is achieved through three key components: (1) the Edge-Enhanced Channel Attention (EECA) module dynamically links edge features of woodland-cropland boundaries with channel semantic weights, thus enhancing the detection of fine-grained boundaries such as forest edges and ridges while suppressing deep semantic noise; (2) the HiLo hierarchical dynamic adaptation strategy allocates differentiated parameters based on the resolution and semantic differences in encoder layers, effectively balancing detail retention and semantic focus; (3) the Dual-Gated Feature Modulation (DGFM) module dynamically filters redundant features from dual temporal phases and amplifies change signals, significantly reducing false detections caused by spectral variations and shadow interference. Collectively, these components establish a precise feature representation and temporal modeling mechanism, substantially improving the accuracy of woodland-to-cropland change detection.

Experimental results demonstrate that the EHDCD model outperforms traditional CNN, transformer, and other Mamba-based models in overall performance on the FC-CD and additional remote sensing change detection datasets. Ablation studies further validate the independent value of each module and the rationality of their combined configuration. Although the model maintains the linear computational complexity characteristic of the Mamba architecture, the increase in parameter size and computational overhead compared to the base Mamba model indicates that further optimization may be required for extremely large-scale monitoring scenarios.

This study has two main limitations: (1) the ability to distinguish between true and pseudo changes in spectrally heterogeneous regions, caused by woodland phenological fluctuations and cropland growth cycles, requires further improvement; (2) the increased parameter size and computational complexity may constrain deployment efficiency in large-scale operational environments. Future research will focus on incorporating semantic discrimination mechanisms tailored to woodland-cropland characteristics to enhance pseudo-change filtering accuracy. Furthermore, lightweight techniques such as parameter pruning and quantization will be explored to reduce computational costs. These developments aim to advance the practical application of the model in large-scale woodland-to-cropland dynamic monitoring, contributing technical support to ecological conservation and cropland protection.

## Figures and Tables

**Table 1:** Performance comparison of models on the CLCD dataset. Highest scores are highlighted in red.

	Methon	Rec (%)	Pre (%)	OA (%)	F1 (%)	IoU (%)
CNN-based	FC-EF	36.29	73.34	94.30	48.64	32.14
	FC-Siam-Diff	31.60	72.97	94.04	44.10	28.29
	FC-Siam-Conc	45.22	68.21	94.35	54.48	37.35
	SNUNet	72.12	61.81	95.37	66.57	49.84
Transformer-based	BIT	68.58	73.43	95.32	66.10	53.12
	Changer	66.51	68.82	95.27	67.64	51.11
	ChangerFormer	73.13	69.81	95.65	71.43	55.56
Mamba-based	RS-Mamba	73.78	71.27	95.84	72.50	76.23
	ChangeMamba	78.71	84.64	97.35	81.57	68.88
	EHDCD	81.28	85.58	97.59	83.37	71.49

**Table 2:** Performance comparison of models on the SYSU-CD dataset. Highest scores are highlighted in red.

	Methon	Rec (%)	Pre (%)	OA (%)	F1 (%)	IoU (%)
CNN-based	FC-EF	72.85	72.28	87.01	72.57	56.94
	FC-Siam-Di	51.45	84.94	86.40	64.08	47.15
	FC-Siam-Conc	71.62	83.03	89.35	76.89	62.45
	SNUNet	74.45	76.49	82.95	77.89	65.03
Transformer-based	BIT	77.61	78.25	89.63	77.93	63.84
	Changer	79.43	71.45	73.95	71.07	55.90
	ChangerFormer	80.27	75.03	81.28	76.71	63.36
Mamba-based	RS-Mamba	77.22	78.93	89.77	78.07	75.76
	ChangeMamba	78.25	87.99	92.35	82.83	70.70
	EHDCD	83.32	86.87	93.10	85.06	74.00

**Table 3:** Performance comparison of models on the FC-CD dataset. Highest scores are highlighted in red.

	Methon	Rec (%)	Pre (%)	OA (%)	F1 (%)	IoU (%)
CNN-based	FC-EF	32.67	72.42	83.03	45.03	29.06
	FC-Siam-Di	55.52	66.12	84.49	60.36	43.22
	FC-Siam-Conc	67.79	59.99	83.53	63.65	46.68
	SNUNet	56.10	73.67	86.40	63.70	46.73
Transformer-based	BIT	55.60	59.00	82.33	57.25	40.10
	changer	74.36	59.29	83.68	65.97	49.23
	ChangerFormer	64.04	67.56	85.81	65.75	48.98
Mamba-based	RS-Mamba	81.43	78.43	91.29	79.90	78.00
	ChangeMamba	86.21	85.91	94.06	86.01	75.52
	EHDCD	90.32	87.39	95.28	89.06	80.28

**Table 4:** Ablation study of EHDCD components on the CLCD and FC-CD datasets.

Model				CLCD				FC-CD			
Base	EECA	HiLo	DGFM	Rec (%)	OA (%)	F1 (%)	IoU (%)	Rec (%)	OA (%)	F1 (%)	IoU (%)
√				78.71	97.35	81.57	68.88	86.21	94.06	86.01	75.52
√	√			78.28	97.44	81.99	69.49	88.07	94.74	87.69	78.08
√	√	√		82.33	97.32	82.04	69.55	87.45	94.99	88.14	78.79
√			√	79.45	97.53	82.69	70.49	87.89	94.94	88.08	78.70
√	√	√	√	83.52	97.54	83.50	71.68	89.70	95.41	89.26	80.61

“√” indicates that the corresponding component is included in the model.

**Table 5:** Comparison of network parameters and computational cost across models (input size: 256 × 256).

	Methon	Params (M)	FLOPs (G)
CNN-based	FC-EF	1.35	3.24
	FC-Siam-Di	1.35	4.39
	FC-Siam-Conc	1.54	4.99
	SNUNet	3.01	11.73
Transformer-based	BIT	2.99	8.75
	Changer	3.45	1.74
	ChangerFormer	3.84	2.46
Mamba-based	RS-Mamba	51.95	22.82
	MambaCD-Small	54.00	30.92
	EHDCD	57.81	31.89

## Data Availability

This study was conducted using public datasets and open-source Google Earth data, with data sources detailed in Section 4.1. For further inquiries regarding the public datasets, please contact the corresponding authors.

## References

[1] Tan Y., Sun K., Wei J., Gao S., Cui W., Duan Y., Liu J., Zhou W. (2024). STFNet: A Spatiotemporal Fusion Network for Forest Change Detection Using Multi-Source Satellite Images. Remote Sens..

[2] Bairwa B., Sharma R., Kundu A., Sammen S.S., Alshehri F., Pande C.B., Orban Z., Salem A. (2025). Predicting Changes in Land Use and Land Cover Using Remote Sensing and Land Change Modeler. Front. Environ. Sci..

[3] Kumar S., Arya S. (2021). Change Detection Techniques for Land Cover Change Analysis Using Spatial Datasets: A Review. Remote Sens. Earth Syst. Sci..

[4] Willis K.S. (2015). Remote Sensing Change Detection for Ecological Monitoring in United States Protected Areas. Biol. Conserv..

[5] Yuan J., Chen E.-Y., Qing H. (2025). A Fast Hyperspectral Change Detection Algorithm for Agricultural Crops Based on Spatial Reconstruction. PLoS ONE.

[6] Ruuhulhaq M.S. (2025). The Role of Remote Sensing and GIS in Sustainable Development and National Resilience. J. Lemhannas RI.

[7] Xu J., Zheng Y. (2024). Remote Sensing Data Analysis for Urban Planning and Land Use Change. Trans. Environ. Energy Earth Sci..

[8] Tasnim S., Mahbub F., Biswas G., Enamul Haque D.M. (2022). Spatial Indices and SDG Indicator-Based Urban Environmental Change Detection of the Major Cities in Bangladesh. J. Urban Manag..

[9] Zhou Y., Li X., Liu Y. (2021). Cultivated Land Protection and Rational Use in China. Land Use Policy.

[10] Popp A., Humpenöder F., Weindl I., Bodirsky B.L., Bonsch M., Lotze-Campen H., Müller C., Biewald A., Rolinski S., Stevanovic M. (2014). Land-Use Protection for Climate Change Mitigation. Nat. Clim. Change.

[11] Uyar N., Uyar A. (2025). Assessing Climate Change Impacts on Cropland and Greenhouse Gas Emissions Using Remote Sensing and Machine Learning. Atmosphere.

[12] Jiang H., Peng M., Zhong Y., Xie H., Hao Z., Lin J., Ma X., Hu X. (2022). A Survey on Deep Learning-Based Change Detection from High-Resolution Remote Sensing Images. Remote Sens..

[13] Caye Daudt R., Le Saux B., Boulch A. Fully Convolutional Siamese Networks for Change Detection. Proceedings of the 2018 25th IEEE International Conference on Image Processing (ICIP), Athens, Greece, 7–10 October 2018.

[14] Fang S., Li K., Shao J., Li Z. (2022). SNUNet-CD: A Densely Connected Siamese Network for Change Detection of VHR Images. IEEE Geosci. Remote Sens. Lett..

[15] Han C., Wu C., Guo H., Hu M., Chen H. (2023). HANet: A Hierarchical Attention Network for Change Detection with Bitemporal Very-High-Resolution Remote Sensing Images. IEEE J. Sel. Top. Appl. Earth Obs. Remote Sens..

[16] Zhou P. Applications of Transformer in Remote Sensing for Image Scene Classification, Semantic Segmentation, and Change Detection. Proceedings of the 2024 2nd International Conference on Computer Science and Mechatronics (ICCSM 2024).

[17] Khan S., Naseer M., Hayat M., Zamir S.W., Khan F.S., Shah M. (2022). Transformers in Vision: A Survey. ACM Comput. Surv..

[18] Chen H., Qi Z., Shi Z. (2022). Remote Sensing Image Change Detection with Transformers. IEEE Trans. Geosci. Remote Sens..

[19] Zheng Z., Ma A., Zhang L., Zhong Y. (2021). Change Is Everywhere: Single-Temporal Supervised Object Change Detection in Remote Sensing Imagery. Proceedings of the 2021 IEEE/CVF International Conference on Computer Vision (ICCV), Montreal, QC, Canada, 11–17 October 2021.

[20] Bandara W.G.C., Patel V.M. (2022). A Transformer-Based Siamese Network for Change Detection. Proceedings of the IGARSS 2022—2022 IEEE International Geoscience and Remote Sensing Symposium, Kuala Lumpur, Malaysia, 17–22 July 2022.

[21] Zhang C., Wang L., Cheng S., Li Y. (2022). SwinSUNet: Pure Transformer Network for Remote Sensing Image Change Detection. IEEE Trans. Geosci. Remote Sens..

[22] Gu A., Goel K., Ré C. (2022). Efficiently Modeling Long Sequences with Structured State Spaces. arXiv.

[23] Ibrahim F., Liu G., Wang G. (2025). A Survey on Mamba Architecture for Vision Applications. arXiv.

[24] Bao M., Lyu S., Xu Z., Zhou H., Ren J., Xiang S., Li X., Cheng G. (2025). Vision Mamba in Remote Sensing: A Comprehensive Survey of Techniques, Applications and Outlook. arXiv.

[25] Chen H., Song J., Han C., Xia J., Yokoya N. (2024). ChangeMamba: Remote Sensing Change Detection with Spatiotemporal State Space Model. IEEE Trans. Geosci. Remote Sens..

[26] Zhao S., Chen H., Zhang X., Xiao P., Bai L., Ouyang W. (2024). RS-Mamba for Large Remote Sensing Image Dense Prediction. IEEE Trans. Geosci. Remote Sens..

[27] Wang T., Bai T., Xu C., Liu B., Zhang E., Huang J., Zhang H. (2025). AtrousMamaba: An Atrous-Window Scanning Visual State Space Model for Remote Sensing Change Detection. arXiv.

[28] Zhang H., Chen K., Liu C., Chen H., Zou Z., Shi Z. (2025). CDMamba: Incorporating Local Clues into Mamba for Remote Sensing Image Binary Change Detection. arXiv.

[29] Lin J., Michailidis G. (2024). Deep Learning-Based Approaches for State Space Models: A Selective Review. arXiv.

[30] Gu A., Gupta A., Goel K., Ré C. (2022). On the Parameterization and Initialization of Diagonal State Space Models. arXiv.

[31] Somvanshi S., Islam M.M., Mimi M.S., Polock S.B.B., Chhetri G., Das S. (2025). From S4 to Mamba: A Comprehensive Survey on Structured State Space Models. arXiv.

[32] Alonso C.A., Sieber J., Zeilinger M.N. (2024). State Space Models as Foundation Models: A Control Theoretic Overview. arXiv.

[33] Smith J.T.H., Warrington A., Linderman S.W. (2023). Simplified State Space Layers for Sequence Modeling. arXiv.

[34] Gu A., Dao T. (2024). Mamba: Linear-Time Sequence Modeling with Selective State Spaces. arXiv.

[35] Liu Y., Tian Y., Zhao Y., Yu H., Xie L., Wang Y., Ye Q., Jiao J., Liu Y. (2024). VMamba: Visual State Space Model. arXiv.

[36] Han D., Wang Z., Xia Z., Han Y., Pu Y., Ge C., Song J., Song S., Zheng B., Huang G. (2024). Demystify Mamba in Vision: A Linear Attention Perspective. arXiv.

[37] Li Y., Xie R., Yang Z., Sun X., Li S., Han W., Kang Z., Cheng Y., Xu C., Wang D. (2025). TransMamba: Flexibly Switching between Transformer and Mamba. arXiv.

[38] Berman M., Triki A.R., Blaschko M.B. (2018). The Lovasz-Softmax Loss: A Tractable Surrogate for the Optimization of the Intersection-Over-Union Measure in Neural Networks. Proceedings of the 2018 IEEE/CVF Conference on Computer Vision and Pattern Recognition, Salt Lake City, UT, USA, 18–22 June 2018.

[39] Liu M., Chai Z., Deng H., Liu R. (2022). A CNN-Transformer Network with Multiscale Context Aggregation for Fine-Grained Cropland Change Detection. IEEE J. Sel. Top. Appl. Earth Obs. Remote Sens..

[40] Shi Q., Liu M., Li S., Liu X., Wang F., Zhang L. (2022). A Deeply Supervised Attention Metric-Based Network and an Open Aerial Image Dataset for Remote Sensing Change Detection. IEEE Trans. Geosci. Remote Sens..

[41] Loshchilov I., Hutter F. (2019). Decoupled Weight Decay Regularization. arXiv.

[42] Fang S., Li K., Li Z. (2023). Changer: Feature Interaction Is What You Need for Change Detection. IEEE Trans. Geosci. Remote Sens..

